# Sensors, Biosensors, and Analytical Technologies for Aquaculture Water Quality

**DOI:** 10.34133/2020/8272705

**Published:** 2020-02-17

**Authors:** Xiaodi Su, Laura Sutarlie, Xian Jun Loh

**Affiliations:** ^1^Institute of Materials Research and Engineering, Agency for Science, Technology and Research, 2 Fusionopolis Way. Innovis #08-03, Singapore 138634; ^2^Department of Chemistry, National University of Singapore, Block S8, Level 3, 3 Science Drive 3, Singapore 117543

## Abstract

In aquaculture industry, fish, shellfish, and aquatic plants are cultivated in fresh, salt, or brackish waters. The increasing demand of aquatic products has stimulated the rapid growth of aquaculture industries. How to effectively monitor and control water quality is one of the key concerns for aquaculture industry to ensure high productivity and high quality. There are four major categories of water quality concerns that affect aquaculture cultivations, namely, (1) physical parameters, e.g., pH, temperature, dissolved oxygen, and salinity, (2) organic contaminants, (3) biochemical hazards, e.g., cyanotoxins, and (4) biological contaminants, i.e., pathogens. While the physical parameters are affected by climate changes, the latter three are considered as environmental factors. In this review, we provide a comprehensive summary of sensors, biosensors, and analytical technologies available for monitoring aquaculture water quality. They include low-cost commercial sensors and sensor network setups for physical parameters. They also include chromatography, mass spectrometry, biochemistry, and molecular methods (e.g., immunoassays and polymerase chain reaction assays), culture-based method, and biophysical technologies (e.g., biosensors and nanosensors) for environmental contamination factors. According to the different levels of sophistication of various analytical techniques and the information they can provide (either fine fingerprint, highly accurate quantification, semiquantification, qualitative detection, or fast screening), we will comment on how they may be used as complementary tools, as well as their potential and gaps toward current demand of real-time, online, and/or onsite detection.

## 1. Introduction

### 1.1. Aquaculture Industry and Challenges

Aquaculture is the farming (breeding, raising, and harvesting) of aquatic organisms, especially for human consumption. It is a global industry with increased importance in battling the challenges of the food supply in the future [1]. The aquaculture industry has, in the last four decades, grown at a rate of 7% on average each year [2], being faster compared to other sectors in animal food production industry. The global human population will eat 30 million tons of fish by 2030, according to the United Nations Food and Agriculture Organization. Millions of people globally have found income and livelihood in the fisheries and aquaculture sector [2].

In aquaculture industry, fish, shellfish, and aquatic plants (such as algae, seaweeds) are cultivated in fresh, salt, and brackish waters. Feeds and feeding, fish health and disease management, good aquaculture practices, etc. are key challenges that affect farm productivity and quality. The lack of understanding in aquaculture nutrition, feed preparation, and proper feeding management will cause less desirable water quality in both the land-based and nonland-based farms due to accumulation of undigested food. Poor disease management, partially due to the slow pathogen identification relying on laboratory culture plate count, and thus improper usage of drugs, will lead to drug/chemical residual deposits in the fish tissue. This will not only pose potential health hazard to humans when consumed, it would also lead to discharge of fish wastes containing such residual chemicals into the surrounding water, causing a buildup of antibiotic or drug resistance in the farmed products and surrounding ecosystem overtime. In offshore aquaculture, or open ocean aquaculture, on the other hand, organic contaminants, like polycyclic aromatic hydrocarbons (PHAs) and polychlorinated biphenyls (PCBs) etc., formed due to incomplete, but high-temperature and short-duration combustions of organic matters including fossil fuels and biomass are another important factor affecting for fish health and quality [3].

To ensure successful scaling up of aquatic farming, water quality control is a key aspect of fisheries management. Technologies capable of rapid, real-time, and automatic monitoring of aquaculture environment are in high demand.

### 1.2. Scope of This Review

**Figure 1:**
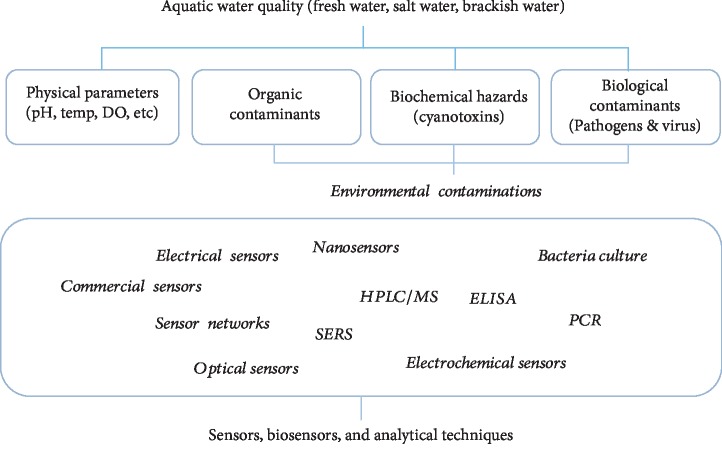
Scopes of this review. Four major water quality parameters and analytical technologies involved. SERS: surface-enhanced Raman spectroscopy; PCR: polymerase chain reaction; ELISA: enzyme-linked immunosorbent assay; LPS: lipopolysaccharides; DO: dissolved oxygen.

In this review article, we will discuss four major areas of aquaculture system monitoring, concerning water quality (Figure 1), namely, physical parameters, i.e., pH, temperature, dissolved oxygen, and salinity (Section 2), organic chemical contaminants (Section 3), biochemical hazard, i.e., cyanotoxins (Section 4), and biological contaminants, i.e., bacteria and virus (Section 5). We will discuss the conventional and modern analytical technologies that have been developed and applied for these parameters and analytes, and their status of commercial exploitation. For newer biosensors and analytical technologies for the environmental contamination factors, we will particularly comment on their strengths and limitations, and their suitability for fast inspection and/or for accurate diagnosis, in either onsite manner or real-time. With a summary of future demand of real-time continuous detection, smart sensors, data connectivity, etc., we hope this review can provide a clear view on the level of readiness of various analytical technologies to meet the future demands.

This review will complement to earlier review articles of relevant scopes, for example, those discuss direct fish health monitoring (stress level, prawning prediction, and fish disease) [4, 5], that for aquaculture pathogens [6], that about sources of monitoring environmental exposure to polycyclic aromatic hydrocarbons [7], and that about detection of cyanotoxins in freshwater [8].

## 2. Physical Parameters

### 2.1. Major Physical Parameters and Their Impact

Physical parameters, including dissolved oxygen (DO), temperature, pH level, salinity, and turbidity, are the basic parameters to be monitored and controlled in an aquaculture system [9]. Fluctuations in these parameters will directly affect the health of the animals, feed utilization, growth rates, and carrying capacities [10].

The temperature of the water affects the feeding pattern and the growth of fish. When the temperature is chronically near their maximum tolerance or fluctuates suddenly, fishes would generally experience stress and disease breakout. Moreover, warm water carries less DO than cool water. The level of DO in water, and hence the amount of oxygen consumed, is directly linked to the size of fish, feeding rate, activity level, and pond temperature [11]. The DO level would decrease as temperature increases, and when salinity increases. Not only the optimal level of DO is essential for fish respiration, it is also imperative for the survival of phytoplankton—an organism that breaks down toxic ammonia into harmless forms. This organism thus plays an indirect part in maintaining the pH level of the water as well. The acceptable range of pH for fish culture is usually between pH 6.5 and 9.0. When pH level is higher than 9, ammonium (NH_4_^+^) in the water is converted to toxic ammonia (NH_3_), a compound that is lethal to fishes. On the other hand, when the pH level goes below 5, acidic water would leach metals from rocks and sediments. These metals would adversely affect fishes' metabolism rates and their ability to take in water through their gills, resulting in a fatality as well.

### 2.2. Wireless Sensor Networks for Physical Parameters

Traditionally, water quality fish farms are measured periodically onsite using handheld sensors. The aforementioned physical parameters are commonly required to maintain acceptable levels for fish growth regardless the type of fishes. While handheld instruments or sensors can provide onsite measurement by the staff during office hours, the variation of one of the key water parameters beyond a safe level can occur out of office hours, unseen by the staff. When the bad situation is persistent, it will lead to undesirable effects, such as poor growth, undetected disease symptoms, or abnormal behavior of the fish [12, 13].

In recent years, advancements in different Information and Communication Technologies (ICT) along with the development of low-cost small sensors have increased the feasibility of monitoring numerous parameters concurrently through wireless sensor networks (WSN). The applications of WSN include the monitoring of the three vigor [14], greenhouses [15], citrus crops [16], the state of farm animals (goats [17] or cows [18]), and fish farms [19–25]. WSN is composed of many self-organized sensors deployed in a monitoring region that measure, collect, transmit, and process information in real time. The information measured is then displayed on a computer or conveyed in the form of a message to farmers for the real-time update. This real-time remote monitoring technology allows the streamlining of the information accumulation process, which conceivably minimizes human lapses and time delays, hence increasing the quantity and quality of data on temporal and spatial scales [20].

Numerous studies have been conducted to study and develop WSN for use in aquaculture [21–25]. Most of these technologies boost the ability to measure the majority of the important physical parameters in real-time and the capability of informing the necessary people at the facility whenever a problem arises, allowing for quick and immediate resolution. Espinosa-Faller and Redón-Rodríguez [22] presented a WSN-based water monitoring system that transmits the gathered information and stores them in a database. The system is also able to measure temperature, pressure, and DO throughout the day. When a problem was detected, an SMS or an E-mail was forwarded to alert the person responsible for the facility. Zhang et al. [23] proposed another WSN-based water monitoring system that was able to measure pH, water temperature, water level, and DO. The data collected was forwarded to a database that provided the information to the software to be monitored in real time. In their system, the software used was able to separate logic, display, and data layers to improve scalability and reusability. Warnings could also be forwarded via SMS to the users. Lastly, Huang et al. [24] presented a WSN-based system that gathered data on pH, temperature, and DO. It contains a real-time interface that displays the data numerically and graphically. More recently, Luo et al. [25] reported a real-time remote monitoring system for aquaculture water quality using solar cells and lithium cells for power supply. In their system, they have integrated the commercial YCS-2000 DO sensor, pH electrode, Pt1000 temperature sensor, and ammonia nitrogen sensor.

**Figure 2:**
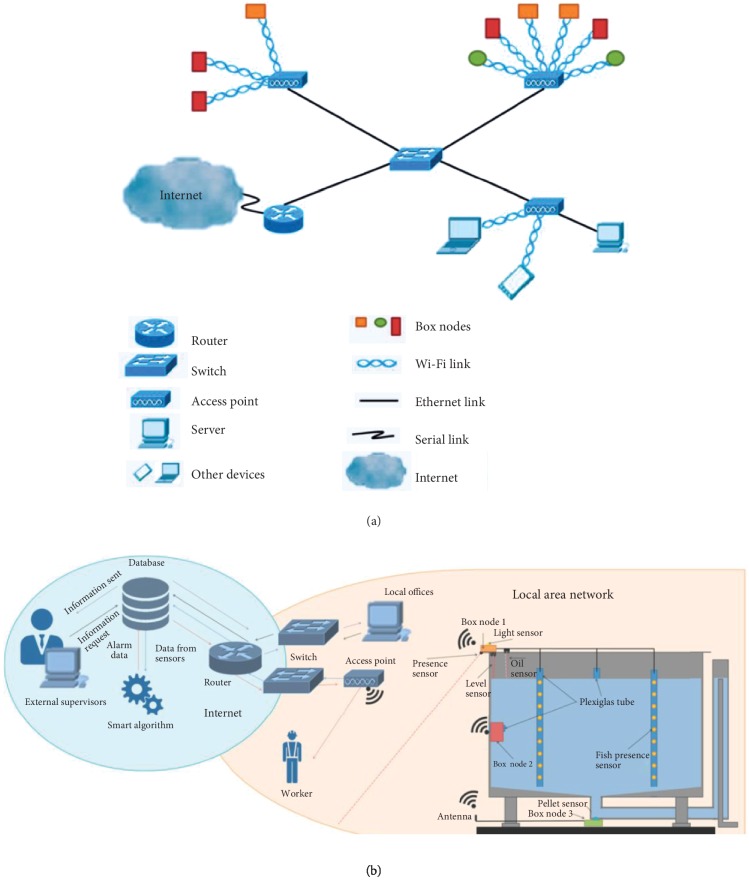
A low-cost sensor network for monitoring the water quality and fish behavior in aquaculture tanks. (a) Network topology. (b) Architecture of proposed system [26] (Open Access).

Not only for water quality monitoring, Parra et al. [26] proposed a WSN for monitoring the water quality and fish behavior in aquaculture tanks during the feeding process (Figure 2). The system is composed of three sensor nodes in each tank that send the information though the local area network to a database on the Internet. A smart algorithm is also included in the system that detects abnormal values and sends alarms when they happen. It is a low-cost system (<90€) including the sensors and nodes.

## 3. Organic Contaminants

Apart from physical parameters that require constant monitoring to optimize water quality in aquaculture, various contaminants (including chemical, biochemical, and biological hazards) from the environment are also of serious concerns of water quality. In this section, we will discuss organic contaminants and their analysis.

### 3.1. Organic Contaminants and Health Risks

Organic compounds, such as polycyclic aromatic hydrocarbons (PAHs), polychlorinated biphenyls (PCBs), organochlorinated pesticides (OCPs), brominated flame retardants (BFRs), and residues of veterinary drugs and antibiotics, can enter aquaculture systems via feed, which can be potentially transferred to organisms [27]. These contaminants are able to bioaccumulate as they go across the food chain, causing detrimental effects on human health upon ingesting contaminated organisms. They also interfere with the food safety of aquaculture products. For example, DDT (dichlorodiphenyltrichloroethane) and aldrin (examples of OCPs) have the strongest tendency to bioaccumulate in fish fatty tissue, eventually reaching to high levels at the end of the food chain [28]. Studies have reported the presence of OCPs in human lipid tissues and human breast milk, of which fish consumption would be a possible source [29–31].

### 3.2. Laboratory-Based Techniques for Organic Contaminant Detection

Sensitive and reliable determination of various contaminants in aquaculture systems, particularly in foodstuffs (fish or shellfish), has been largely relied on laboratory based techniques, including liquid (LC) or gas chromatography (GC) combined with mass spectrometry (MS). GC is preferred over LC due to its higher selectivity and resolution for complex matrices such as aquaculture samples [32]. Configurations, such as GC coupled to tandem MS with a triple quadrupole (QqQ), successfully detected 16 PAHs which were present in the Environmental Protection Agency (EPA) priority pollutant list. GC–ECD (electron capture detection) was used to measure PCBs [33], while OCPs were measured by GC-MS. Finally, UHPLC-MS/MS was widely used to simultaneously determine 41 antibiotics [34].  Table 1 is a summary of these studies, from which one can have a clear view on the specific sample types and the performance of these methods. For organic contamination analysis in solid aquatic samples (e.g., fish body, tissue, fish fillet, and fish oil), microwave-assisted extraction (MAE), conventional solvent microextraction, and ultrasound-assisted extraction (UAE) techniques have been largely developed and applied to extract the analytes [34, 35]. In two most recent review articles, MAE and UAE techniques have been discussed for their working principle, efficiency, and applications for both solid and liquid sample extractions in food and environmental samples [36, 37].

### 3.3. Biochemistry Methods

The enzyme-linked immunosorbent assay (ELISA) is a very popular analytical biochemistry method. It has been widely developed for various analytes from macromolecules (e.g., proteins and DNA) to small-molecular-weight drugs and other organic compounds of health and environmental concerns. ELISA mostly relies on antibodies to capture respective target analytes in microplate wells. After a few steps of incubation and washing, immunocomplexes are formed inside the wells. The analytes are detected by either colorimetric or fluorescent signals generated by the enzyme-conjugated antibody in the complexes in the presence of enzyme substrates.

There are a range of ELISA kits available for PAHs, PCBs, and antibiotic residues.  Table 2 shows a few examples that are mostly for water and soil analysis. Since these analytes are mostly small molecules, it is difficult to use the sandwiched assay principle, but more of competitive principles. The assays can be in either microplate format or using magnetic beads. Antibodies against the chemical and chemical groups are used as the major affinity ligand to bind with the chemicals.

ELISA is considered more portable than GC-MS and HPLC, due to the reliance of less sophisticated plate reader. But they still require hours of incubation and multiple washing steps. Moreover, since antibodies often have the cross reactivity to chemicals of similar structure, the assays usually cannot differentiate individual PHAs, for example, as stated by the RaPID Assay® and Aviva PAH ELISA Kit. Sensitivity wise, ELISA offers similar sensitivity as GC-MS. ELISA can serve the purpose of fast screening. Those ELISA-positive samples are usually sent for HPLC and GC-MS for confirmation.

### 3.4. Biosensors and Nanosensors

**Figure 3:**
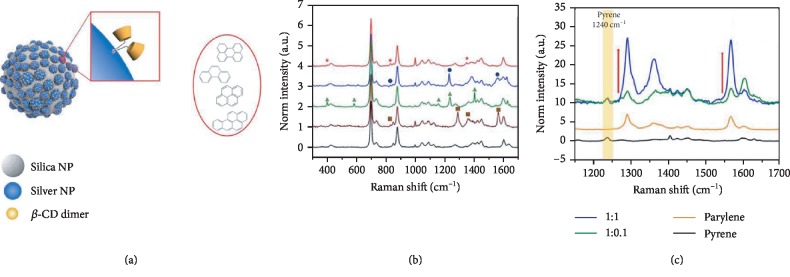
(a) Schematic of *β*-CD dimer-immobilized Ag assembly with embedded silica NPs (*β*-CD dimer@Ag@SiO_2_ NPs) for SERS detection of PAHs. (b) SERS spectra of four PAHs. (c) SERS spectra of perylene and a fixed concentration of pyrene [44] (Copyright © 2020, Springer Nature).

Nanomaterials and nanotechnologies have been largely applied in analytical sciences. Metal nanoparticle-based surface-enhanced Raman spectroscopy (SERS) is a powerful technique to provide fingerprint information for trace chemicals. It has been largely applied for identification and detection of organic contaminants in water, particularly PAHs, e.g., phenanthrene and fluorene [40], naphthalene and pyren [41], anthracene and pyrene [42], and the mixture of PAHs [43, 44]. In these studies, metallic nanostructures were functionalized by hydrophobic films (e.g., glycidyl methacrylate-ethylene dimethacrylate, 10-decanethiol monolayer, or *β*-cyclodextrin) to allow preconcentration of nonpolar molecules on the nanostructure surface. The chemical identities and concentration of each chemical are analyzed by their distinct Raman signatures, amplified under the plasmonic effect.  Figure 3 shows the example where *β*-CD dimer on silver nanoparticles embedded with silica nanoparticles (Ag@SiO_2_ NPs) structure is fabricated for detecting PAHs [44]. A thioether-bridged dimeric *β*-CD was immobilized on Ag surface to capture PAHs. The *β*-CD dimer@Ag@SiO_2_ SERS sensor can detect perylene in a wide linearity range of 10^−7^ M to 10^−2^ M with a low detection limit of 10^−8^ M (1000 times lower than that without *β*-CD dimer). Furthermore, this *β*-CD dimer@Ag@SiO_2_ SERS sensor and another gold nanoparticle/*β*-CD sensor [43] can detect multiple PAH compounds in a mixture by exploiting their distinct SERS bands. In another gold nanoparticle-based SERS study, its ppb level sensitivity for PHAs and the potential for field test has been demonstrated through offshore experiments [41].

## 4. Biochemical Hazards

### 4.1. Biochemical Hazards and Health Risks

With increasing global climate change and eutrophication, there has been a rise in Harmful Algal Blooms (HABs), exponential blooming of algae, in freshwater and marine ecosystems [45–47]. Some cyanobacterial species of HABs produce cyanotoxins that usually target human nervous systems (neurotoxins), livers (hepatotoxins), or skin (dermatoxins). The chemical structure of cyanotoxins falls into three broad groups: cyclic peptides, alkaloids, and lipopolysaccharide (LPS) endotoxins. They are nature-occurring organic pollutants.

Cyanobacterial hepatotoxins, such as a group of toxic cyclic peptides called microcystins (MCs) with ~80 congeners, nodularin (NODs), and cylindrospermopsin (CYN), are known to cause liver failure [48]. MCs are reported in numerous regions such as Asia, Europe, North Africa, North America, and Scandinavian countries, while NODs are confined within Australia, New Zealand, and the Baltic Sea [49]. Evidence of accumulation of these toxins in humans from fish in MC-contaminated waters is documented by a study conducted on fishermen subsisting from Lake Chaohu in China, where MCs were detected in the serum of fishermen [50].

Lipopolysaccharide (LPS) endotoxins are structural components of the outer membrane of cyanobacteria and other gram-negative bacteria. They are responsible for a wide range of infections in humans, including sepsis and septic shock. LPS endotoxins are found in all types of water including freshwater, saline surface water, and groundwater.

### 4.2. Conventional Analytical Technologies for Biochemical Hazards

Conventional methods for cyanotoxins analysis are chromatography tandem mass spectrometry [51–53]. For example, an ultra-performance liquid chromatography tandem mass spectrometry (UPLC-MS/MS) method was developed to measure nine cyanotoxins in fish muscle tissue, and free cyanotoxin levels in 34 fish (muscle tissue) and 17 livers and eggs from five pond-based aquaculture farms in Southeast Asia [46]. The results indicated the presence of cyanotoxins. The levels are lower than those in Zaria, Northern Nigeria, and China, but similar to those reported from a freshwater lake in Mexico. While being highly accurate and sensitive, these technologies require sophisticated laboratory equipment and are not suitable for onsite rapid analysis.

### 4.3. Biochemistry Methods for Biochemical Hazards

There is a diverse range of biochemistry methods that are considered as rapid tests to detect and identify cyanobacteria cells and cyanotoxins in water, including enzyme-linked immunosorbent assays (ELISA), protein phosphatase inhibition assay (PPIA), protein synthesis inhibition assay (PSI), conventional polymerase chain reaction (PCR), quantitative real-time PCR (qPCR), and microarrays/DNA chips [54]. These methods can vary greatly in their degree of sophistication and the information they provide. For example, the PPIA is a cost-effective assay using p-nitrophenyl phosphate to quickly assess water and rumen content samples [55]. Researchers have compared this rapid assay with LC-MS. They have concluded that this rapid assay can be used in combination with LC-MS, where after the PPIA, subsequent analysis using liquid chromatography mass spectrometry (LC-MS/MS) can provide more detailed information about microcystin congeners -LR, -LA, -RR, and -LF. They have commented on the advantages of using this rapid functional assay in combination with LC-MS/MS. Similar to the PPIA, PSI quantify cyanotoxins by nonphosphatase-related protein inhibition. Froscio et al. conducted PSI for cyanobacterial toxin cylindrospermopsin (CYN) quantification [56]. The results were compared to quantifications obtained by HPLC and HPLC-tandem mass spectrometry (HPLCMS-MS). They found that the results correlate well with both HPLCMS-MS (*r*^2^ = 0.99) and HPLC (*r*^2^ = 0.97) quantifications. While PSI and PPIA are mostly for cyanotoxins detection, PCR and qPCR are nucleic acid-based technologies mostly for identifying cyanobacteria (more PCR-related bacteria detection is in Section 5).

In a comparative cell numbers and toxin concentrations measured using ELISA, it is found that the results do not necessarily correlate and that enumeration of potentially toxic cyanobacteria by microscopy. The concentrations of certain cyanotoxins (e.g., saxitoxins) quantified by ELISA were significantly different than those measured by LC-MS. On the other hand, results were comparable in both assays for other cyanotoxins (e.g., microcystin and cylindrospermopsin). With all these facts, people have reached to a conclusion that there is no “gold standard” technique for the detection of cyanotoxins. The choice of a detection assay depends on cost, practicality, and reliability of the results that can indicate toxin exposure potential [54].

### 4.4. Biosensors for Cyanotoxins and Cyanobacteria

Biosensors are compact analytical devices and have been largely developed for toxin and bacteria analysis [57–59]. They could be more appealing for onsite and real-time measurement [60]. Among various biosensor formats, optical and electrochemical sensors are mostly developed for aquatic water analysis. Cunha et al. reported an optical sensor that exploits DNA aptamer and quantum dots (QDs) for real-time onsite detection of aquatic toxins produced by marine and freshwater microorganisms (cyanobacteria, dinoflagellates, and diatoms) [61]. This sensor exploits fluorescence resonance energy transfer (FRET) between a quencher-labeled DNA aptamer and QDs, in the presence of target toxin.

Lebogang et al. [62] reported an electrochemical, particularly capacitive immunosensor for broad-spectrum detection of the group of toxic cyclic peptides, called microcystins (∼80 congeners). The sensor can detect at very low concentration range (1 × 10^−13^ M to 1 × 10^−10^ M) closer to the MC-LR standard, with a limit of detection of 2.1 × 10^−14^ M. Yu et al. [63] presented an electrochemical sensor for microcystin-LR that exploits a quantum dot/antibody (QD/Ab) probe for signal amplification. A qualitative analysis for microcystin-LR was achieved using the specific peak potential of the anodic voltammogram at −0.6 ± 0.05 V. The microcystin-LR analyte can be detected in a dynamic range of 0.227 to 50 *μ*g/l with a limit of detection of 0.099 *μ*g/l.

**Figure 4:**
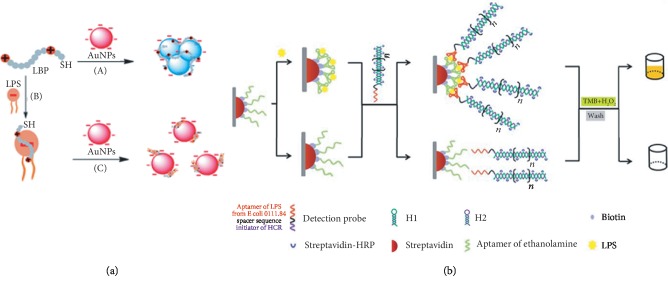
(a) Colorimetric LPS sensor exploiting gold nanoparticle aggregation [66]. (b) Hybridization chain reaction-based aptasensor for LPS [76] (Open Access).

Due to the wide range concern of endotoxin contamination, LPS biosensors have been largely developed, including optical fiber-based plasmonic sensor [64], colorimetric sensors [65, 66], electrochemical sensors [8, 67, 68], and nanomaterial sensors [69]. The nanomaterial-based LPS sensors have exploited versatile sensing principles, where the nanoparticle either being used as direct signal transducer, i.e., colorimetric sensing based on their aggregation (Figure 4(a)) or as signal amplifier in electrochemical sensors [62, 68]. Metal nanoparticle aggregation-based colorimetric sensors are easy to use (mostly with simple mixing, followed by either visual inspection of color change or UV-vis measurement of the absorption spectrum) [70–72]. But they are prone to generate fault-positive results as they tend to aggregate in complex sample matrix. Thus, a careful design of the sensor aggregation strategy to avoid fault aggregation and a careful study of the sample matrix effects on the aggregation are essential [73–77].

To achieve an ultrahigh sensitivity, Xie et al. developed a colorimetric aptasensor based on DNA hybridization chain reaction (HCR) in microplates (Figure 4(b)) [78]. Briefly, two complementary biotinylated DNA hairpins coexisted in the assay solution. They will not hybridize until the introduction of a detection probe. The detection probe consists of three regions, namely, LPS-binding aptamer, a spacer, and HCR initiator. In the presence of LPS, this detection probe triggered a hybridization chain reaction cascade in the microplate, where the LPS were captured by the ethanolamine aptamer attached to the reaction well surface. Under the optimal conditions, the increase in the LPS concentration led to increase of the optical density value generated by the HRP-catalyzed color reaction. The sensor has a LOD of 1.73 ng/ml and a linear response range of 1–10 ng/ml.

**Figure 5:**
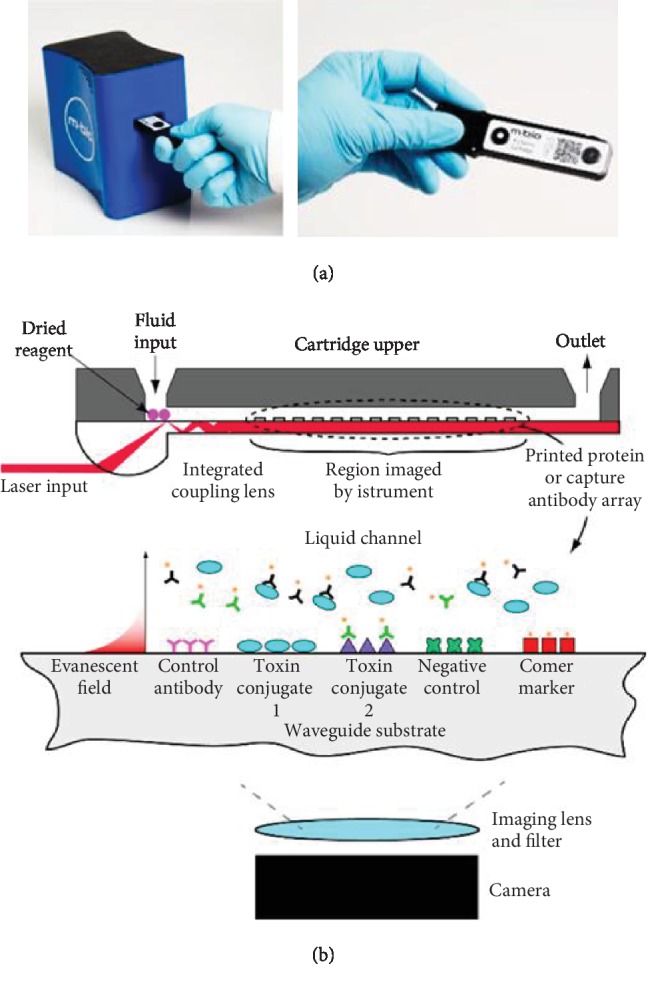
A portable planar waveguide optical sensor for rapid detection of freshwater cyanotoxins. (a) The proposed MBio reader and cartridge. (b) Schematic of LightDeck technology elements [77] (Copyright © 2020, American Chemical Society).

As mentioned in Section 2, portable sensors for water physical parameters have been largely available. But for environmental contaminants, sensor portability has been less well addressed. Dickman et al. recently reported a portable biosensor system for rapid detection of freshwater cyanotoxins [79]. It is a planar waveguide optical sensor that delivers quantitative fluorescent competitive immunoassay results in a disposable cartridge. They supply a duplex microcystin (MC)/cylindrospermopsin (CYN) assay cartridge that relies on a combination of fluorophore-conjugated monoclonal antibodies (Figure 5). More of this type of portable biosensor is in great demand in aquatic industry.

In addition to the detection of cyanobacteria toxins, direct detection of cyanobacteria is also useful for water quality assessment. A nucleic acid biosensor assay has been described to detect cyanopeptolin coding region of one of the cyanobacteria (*Planktothrix agardhii* NIVA-CYA 116) genome for monitoring of the fresh water resources [78]. This is an electrochemical sensor integrated to a microfluidics system. A real-time amperometric measurement leads to a detection limit of 6 × 10^−12^ M target DNA (calibration curve *r*^2^ = 0.98).

## 5. Biological Contaminants

### 5.1. Fish Pathogens

A pathogen is defined as an organism that causes diseases to its host. Pathogens are widely diverse, including mainly bacteria and viruses [80]. They damage animal tissues or cells during replication, usually by generating toxins, allowing pathogens to enter new tissues or exit the cells inside which they replicated. Fish and fish product-related bacteria and virus are diverse. Based on the extent of infection, some of them only infect or kill fishes without infecting human, e.g., *Vibrio harveyi*, *Vibrio anguillarum*, and *Aeromonas salmonicida* [81, 82], but some of them can infect both fish and human such as *Vibrio vulnificus*, *Mycobacterium marinum*, and *Streptococcus iniae* [83]. There are also pathogens that may not necessarily infect the fish, but infect human through consumption of contaminated fish products, e.g., *Vibrio cholerae*, *Escherichia coli*, and *Salmonella* spp.

Among many bacteria pathogens, *Vibrio* spp., a genus of gram-negative bacteria ubiquitous in many aquacultures and marine habitats, are the most common and serious pathogens in fish and shellfish aquaculture worldwide [81]. Many of their species, such as *Vibrio vulnificus*, *Vibrio harveyi*, *Vibrio anguillarum*, *Virbio alginolyticus*, *Vibrio parahaemolyticus*, and *Vibrio salmonicida*, cause mass death and vibriosis (marked by infection on skin and other organs) in many cultured fish, shrimps, and shellfish [81, 84]. Among the *Vibrio* spp., around 12 species of them cause infections in humans, which can be classified into cholera (by *Vibrio cholerae)* and noncholera *Vibrio* infections (by *Vibrio parahaemolyticus*, *Vibrio alginolyticus*, and *Vibrio vulnificus*) [85].

Analytical methods for quantitative and qualitative pathogen detection in aquaculture include culture-based methods, molecular methods, biosensors, and microscopy observation methods. The first three methods will be discussed in the following sessions due to their popularity (e.g., culture methods are considered as current standard), power of quantitative and qualitative analysis (e.g., molecular methods), and high potential for onsite and real-time analysis (e.g., biosensors).

### 5.2. Culture-Based Methods

**Figure 6:**
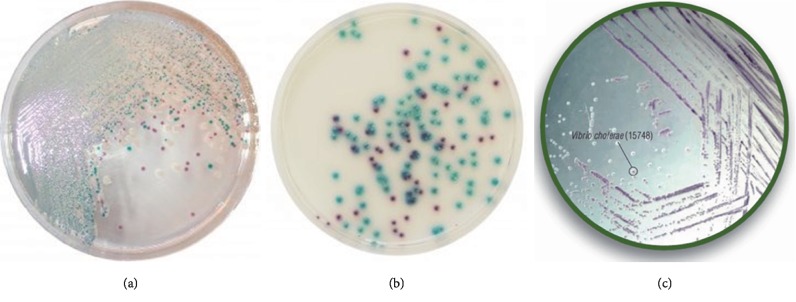
Plate culture for isolation and detection of (a) *Vibrio* using CHROMagar™ Vibrio, (b) *Vibrio* using HiCrome Vibrio Agar (Merck), and (c) *Pseudomonas* spp. using CHROMagar™ *Pseudomonas*.

Culture-based methods are considered as current “standard” for bacteria pathogen detection. In culture methods, bacteria samples from aquaculture water or part of the infected fish are incubated on agar medium and the bacteria colonies grown on the agar are enumerated. Various agar media have been formulated to culture fish pathogens, including general media to culture a broad range of potential pathogens, and specialized media (selective/differential) to target specific pathogens. The specialized media are typically formulated with selective agents to promote growth of target bacteria, inhibitor agents to stop the growth of other bacteria, and color indicator to differentiate target bacteria [86]. A few commercial media for fish pathogens are shown in Figure 6.

Culture-based methods have limitation as they are time-consuming (more than 1 day to get results), not suitable to grow bacteria in viable but nonculturable state, and require lab setting. In addition, specialized media may not be able to completely identify the target pathogens at species and strain level, and further pathogen identification would require more specific molecular methods or biosensors [87].

### 5.3. Molecular Assays-PCR

Bacteria and virus identification and detection can be at cell level or molecular level. DNA is currently the best molecule for bacteria detection as it presents evident biological information. Polymerase chain reaction (PCR) is a popular technique extensively employed to amplify a single or several copies of a specific DNA sequence present in a heterogeneous population to millions of copies with high precision in a short duration. It can detect a large variety of bacteria. Several PCR variants have been developed, such as reverse transcriptase PCR, real-time PCR (RT-PCR), real-time revers transcription PCR (qRT-PCR), and multiplex PCR and nested PCR to identify bacteria and viruses with high accuracy. Among all variants, RT-PCR is most powerful. It allows simultaneous monitoring of the formed product as amplification proceeds. Fluorescent dyes or fluorescent probes are utilized to visualize the amplified product. For example, *Norovirus* detection by qRT-PCR [88] shows improved specificity and sensitivity. It covers all known human NoV genotypes with the use of only four forward primers, two probes, and one reverse primer.

The power of PCR for simultaneous identification of multiple marine fish pathogens (i.e., the multiplex PCR) has been reported. For example, multiplex- (m-) PCR-based protocol was designed for the simultaneous detection of the main marine bacterial pathogens in Chilean salmon farms: *Streptococcus phocae*, *Aeromonas salmonicida*, *Vibrio anguillarum*, and *Piscirickettsia salmonis* [89]. Each of the 4 oligonucleotide primer pairs exclusively amplified the target gene of the specific bacterial pathogen. With the adequate sensitivity for the multiple pathogen (50 pg *μ*l^−1^ for *V. anguillarum*, 500 fg *μ*l^−1^ for *P. salmonis*, and 5 pg *μ*l^−1^ for *S. phocae* and *A. salmonicida*), this technique is considered as an alternative to culture-based methods for the diagnosis of infections in fish. In general, PCR technique can identify and detect bacteria directly from a water sample with or without preenrichment. It is faster (hours) than the culture plate count (days).

### 5.4. Biosensors

**Figure 7:**
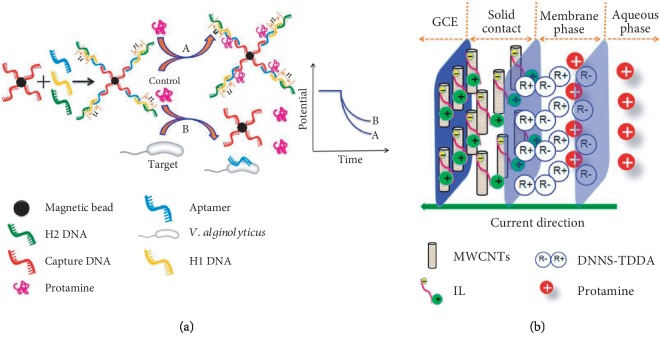
A potentiometric aptasensing of *V. alginolyticus* based on DNA nanostructure-modified magnetic beads. (a) Schematic illustration of the principle. (b) Schematic diagram of the polycation-sensitive electrode based on a MWCNT-IL composite as a solid contact for the chronopotentiometric detection of protamine [98] (Open Access).

Biosensors have been developed for detection of pathogens related to livestock and poultry [90] and for *Vibrio cholerae* [91].  Table 3 is a summary of various biosensors for detecting fish pathogens. They include quartz crystal microbalance (QCM), the microcantilever sensor, amperometric sensor, potentiometric sensor, and surface plasmon resonance (SPR) biosensor, and lateral flow tests, targeting either viral RNA [92–94] or the bacteria cells [95–99]. Biosensors are typically designed to detect known bacteria or virus, but less so for identification of unknown. In a typical biosensor design, DNA probe or antibodies are immobilized on the sensor surface to capture target analyte of viral RNA or bacteria cells, respectively. While biosensors are considered more suitable for onsite application, the RNA detection poses two major limitations, namely, (1) requiring sample preparation to extract the RNA and (2) involving PCR processes. On the other hand, direct detection of bacteria cells by biosensors does not require extraction step and could be more suitable for onsite application so long as the sensitivity can fulfill the requirement. Lateral flow test can be considered as the most portable form of biosensor that combines sample separation, interaction, and detection in one chromatographic strip. For *Vibrio cholerae* O1, for example, a lateral flow test can reach to a LOD of 10^7^ cfu/ml. To push to a lower detection limit of 10^2^ cfu/ml, 6 hours of culture enrichment is needed. The SPR sensor work can be appreciated by its effort of screening aptamer for *Vibrio parahaemolyticus* and the decent selectivity of the aptamer to this specific bacteria, relative to a few others, *E. coli*, *L. monocytogenes*, *V. fischeri*, and *S. soneii*. However, most of the SPR equipment is still a bulky laboratory-based setup that is not suitable for onsite applications [100–102]. In the context of using aptamer as sensing probe, Zhao et al. developed a potentiometric aptasensor for *Vibrio alginolyticus*, involving DNA nanostructure-modified magnetic beads [98]. In this design (Figure 7), the bacteria cells compete the DNA aptamer with the DNA-coated magnetic bead, causing disassembly of the DNA nanostructures on the bead. The change in the charge or DNA concentration on the magnetic beads is chronopotentiometrially detected by a solid-contact polycation-sensitive electrode using protamine as an indicator based on the electrostatic interaction between DNA and protamine. With this method, *Vibrio alginolyticus* can be detected within a linear range of 10–100 cfu/ml and with a LOD of 10 cfu/ml. This proposed strategy can be used for the detection of other microorganisms by changing the aptamers in the DNA nanostructures.

In general, among the methods discussed in Section 5 (culture-based method assays, novel molecular methods, and biosensors), the culture-based methods are the “gold standard” in bacteria detection. Molecular-based techniques have a higher sensitivity, but they require specific and expensive equipment. Fast, reliable, easy to use, sensitive, and specific systems are always in great demand for field use to predict outbreaks and during the outbreak. Biosensors would have such potential, subjected to performance improvements.

## 6. Challenges and Future Perspectives

The increasing demand of aquatic products has stimulated the rapid growth of aquaculture industries. However, a healthy growth of this industry sector requires strong technology innovation. Water quality is one of the key determining factors. Freshwater tank high-density farming, seawater offshore farming, and pond farming all face unique challenges in relation to respective water quality factors, e.g., different types of contaminants and the source of contaminants.

Among all four water quality aspects, sensing and detection technologies for basic physical parameters have all been very well developed and available in the market. A quick Google search of *aquaculture water quality monitoring* will lead to the appearance of many companies that provide commercial solutions, including YSI (a xylem brand, USA), Nilebot (Maadi, Cairo, Egypt), and Aquasend (USA), among many others. Most of the products claim features of *Multiparameter*, *Online Analyzer*, *Intelligent Water Quality Controller*, etc. The actual products indeed are capable of online monitoring of several different parameters by selecting corresponding sensors upon different requests, which usually includes temperature, pH, conductivity, DO, turbidity, sludge concentration, chlorophyll, and blue-green algae. These systems have all aimed to address the demand of multimode sensors, creating data connectivity, and feedback loop to trigger actions. A reliable sensor network should come with optimal sampling strategies, to ensure the reliability of the data.

In comparison to low-cost portable physical sensors, sensors for organic chemical, biochemical, and biological hazards are still behind in their feasibility for onsite application without scarifying performance as laboratory methods. In one example, researchers have developed an immunomagnetic separation-coupled laboratory PCR or flow cytometry to reach ultrahigh sensitivity for a fish pathogen [103, 104]. How to couple such or similar sample enrichment techniques with portable sensors for onsite application with sufficient sensitivity for early warming would be an area that requires further R&D. For pathogen detection, PCR method dominates the practice, but it requires sample preparation to extract DNA or RNA, and its performance heavily depends on reagent quality (DNA primers and enzymes etc.) and PCR apparatus.

To bridge the gaps of multiple parameter systems covering a wider range of parameters (physical, chemical, biochemical, and biological), firstly the chemical, biochemical, and biological sensors must reach to the desired level of portability, and then advanced WSN technology would be needed to cover not only the physical parameters but also those hard-to-measure chemical contaminants and biohazards. For all parameters, the sensor technologies must be highly robust because the aquatic environment varies from time to time. The uncontrollable environmental factor may pose challenges to the reliability of the sensing technologies. Also, the sensors and sensor physics must be compatible with seawater for their background and unstable chemical composition. Future prospects of the monitoring system include improving current aquaculture systems and equipment. This can be carried out by miniaturization of the highly robust and accurate autonomous system, but at the same time allowing contaminants and physical parameters to be measured simultaneously at low costs. Such a technology would probably require the combination of nanotechnology, microelectronics, and microfluidics to achieve this cost-effective production. With this advance system, aquaculture industries can get a more comprehensive data/parameters to better control and create optimum condition that maximize fish production even at remote areas. The system shall have a versatile platform, enabling it to adapt to various industries besides aquaculture, which includes tourism and agricultures. In addition, the advance system also will better support sustainable practices such as the prevention of overfishing and using fewer antibiotics to prevent antimicrobial resistance.

## 7. Conclusion

In summary, aquaculture is the fastest growing sector of agriculture in the world, due to the world's growing population and demand for seafood. We have discussed the various analytical technologies and biosensors used within aquaculture to detect and monitor various water parameters (physical, organic chemical, biochemical, and biological) affecting aquaculture cultivations. Numerous analytical techniques are available in literatures and in the markets, with different levels of sophistication and cost. However, the monitoring and control of aquacultures is a complex task, which requires continuous development of technologies and combinatorial application of various technologies. Thus, not one technology can master it all, and knowledge of the advantages and disadvantages of different technologies available is vital. Some future outlooks include miniaturization of an automated system that can simultaneously measure various contaminants and parameters.

## Figures and Tables

**Table 1:** Laboratory-based analytical techniques for organic contaminants in aquaculture samples.

Target analyte	Matrix	Analytical technique	Performance		Ref.
			Dynamic range	LOD	
PAHs	Fish fillet from gilthead sea bream (*S. aurata*)	GC/QqQ-MS/MS	0.2-200 ng/ml	0.02 *μ*g/kg-0.1 *μ*g/kg	[32]
PCBs	PCBs Fish muscle from rainbow trout (*Oncorhynchus mykiss*)	GC–ECD	1–200 ng/ml	0.1 (PCB 105)–1.4 (PCB 153) ng/g	[35]
OCPs	Muscle tissues of five fish species (*O. mossambicus*, *L. parsia*, *E. suretensis*, *C. striata*, and *S. wynaadensis)*	GC-MS	5–200 ppb	0.7–18.2 ng/ml	[38]
Antibiotics	Fish muscle from gilthead sea bream (*S. aurata*)	UHPLC-MS/MS	50–300 *μ*g/kg	NIL	[39]

**Table 2:** Commercial ELISA kits for PHAs, PCBs, and antibiotic residues.

Kit/company	Principle	LOD	Sample matrix
PAH RaPID Assay (Strategic Diagnostics Inc.)	Competition ELISA on magnetic beads	Total PHAs in ppb level	Groundwater, surface water, well water
Total petroleum hydrocarbon (TPH) (HACH)		Soil: 20, 50, 100, 200 ppm as diesel fuel Water: 2, 5, 10, 20 ppm as diesel fuel	Soil and water
Polychlorinated biphenyls (PCBs) (HACH)	Competitive colorimetric ELISA assay	Semiquantitative screening based on thresholds for PCB	Water
MaxSignal® Florfenicol ELISA Test Kit	Competitive colorimetric ELISA assay	0.2-1.0 ppb	Human samples (urine and serum) foods (milk, meat, egg, honey, etc.)
RaPID Assay® ModernWater (UK)	Competitive colorimetric ELISA assay	Soil: 0.2 ppm to 5 ppm as phenanthrene Water: 2.66 ppb to 66.5 ppb as phenanthrene	Soil and water
Aviva PAH ELISA Kit	Competition ELISA microplate	LOD 10 ng/ml	Environmental PAH samples
Abraxis Tetracyclines ELISA	Competition ELISA microplate	4.0 ppb in honey; 4.0 ppb in milk; 8.0 ppb in meat; 4.0 ppb in shrimp; 0.11 ppb in water	Food and water

**Table 3:** Molecular sensors and bacteria cell sensors for fish pathogens.

Biosensor format	Pathogen	Analyte	Performance	Ref.
Quartz crystal microbalance (QCM)	*Viral haemorrhagic septicaemia (VHS)*	Viral RNA	LOD 1.6 nM	[92]
Electrochemistry with gold nanoparticle for signal amplification	*Aphanomyces invadans*	Viral RNA	LOD 0.5 fM of linear target DNA 1 fM for PCR product	[93]
Lateral flow with gold nanoparticle	*Nervous necrosis virus (NNV)*	Viral RNA	LOD 270 pg of PCR product	[94]
Microcantilever	*Vibrio cholerae* O1	Cells	Dynamic range 1 × 10^3^-1 × 10^7^ CFU/ml LOD ~ 1 × 10^3^ CFU/ml	[95]
Lateral flow with AuNPs	*Vibrio cholerae* O1 and O139	Cells	LOD 10^7^ cfu/ml LOD 10^3^ cfu/ml after 6 h culture enrichment	[96]
Amperometric immunosensors	*Vibrio cholerae* O1	Cells	LOD 8 cfu/ml in seawater 80 cfu/ml in sewer water and tap water	[97]
Potentiometric aptasensing involving magnetic beads	*Vibrio alginolyticus*	Cells	Dynamic range:10–100 cfu/ml LOD 10 cfu/ml	[98]
Surface plasmon resonance spectroscopy	*Vibrio parahaemolyticus*	Cells	Not specified	[99]
